# Acceptability of Generative AI-authored Messages for Promoting Physical Activity for Middle-aged and Older Adults

**DOI:** 10.1093/geroni/igaf122.3520

**Published:** 2025-12-31

**Authors:** Allyson Tabaczynski, Yingjia Liu, Saeed Abdullah, Lizbeth Benson, David Conroy

**Affiliations:** University of Michigan, Ann Arbor, Michigan, United States; University of Michigan, Ann Arbor, Michigan, United States; The Pennsylvania State University, University Park, Pennsylvania, United States; University of Michigan, Ann Arbor, Michigan, United States; University of Michigan, Ann Arbor, Michigan, United States

## Abstract

The delivery of digital health interventions for middle-aged and older adults can be streamlined by using generative artificial intelligence (GenAI) to write text message content. Biases in GenAI systems could lead to culturally insensitive or low-quality messages. Evaluating the acceptability of GenAI-authored messages is crucial before use in health interventions. This research examined middle-aged and older adults’ perceptions of the cultural sensitivity and quality of GenAI-authored text messages for promoting physical activity, and the person- and message-level factors influencing these perceptions. In a cross-sectional survey, middle-aged and older adults (i.e., ≥ 40 years of age; N = 630; Mage=56.8 ±10.1 years) read 80 text messages written by GenAI and identified those that were culturally insensitive or had other problems. Descriptive statistics identified the proportion of GenAI-authored messages labeled as having issues. Separate negative binomial regressions examined the participant (zero-inflated) and message factors associated with message issues. Of 49,859 and 49,894 messages sorted to determine cultural sensitivity and quality, only 4.9% and 6.1% of the messages, respectively, were labeled as having issues. Knowledge of AI-authorship and more favorable attitudes towards AI were associated with identifying more messages as culturally insensitive. Messages generated by prompts that targeted sitting less (compared to moving more) or that described preparing for activity (compared to performing physical activity) received more labels as containing quality issues. Overall, GenAI can be prompted to write high-quality, culturally sensitive text messages for promoting physical activity for middle-aged and older adults, but message content and participants’ knowledge of AI use could influence perceptions.

